# Synergistic inhibition of *Pseudomonas fluorescens* growth and proteases activities via sodium chlorite-based oxyhalogen

**DOI:** 10.1007/s11274-022-03471-6

**Published:** 2022-12-05

**Authors:** Assem Abolmaaty, Reham M. M. Abdelkader, Dina H. Amin

**Affiliations:** 1 Department of Food Science, Faculty of Agriculture, Ain Shams University, Cairo, Egypt; 2 Radiation Microbiology Department, National Center for Radiation Research and Technology, Egyptian Atomic Energy Authority, Cairo, Egypt; 3 Department of Microbiology, Faculty of Science, Ain Shams University, Abbasiya, Cairo, 11566 Egypt

**Keywords:** *Pseudomonas*, Salmide, Microbioreactor, Protease, Antimicrobial, Oxy­halogen, Sodium chlorate

## Abstract

*Pseudomonas fluorescens* is considered among the main spoilage microorganisms due to its ability to produce proteases. Food deterioration caused by spoilage microorganisms has a major impact on food quality and the environment. The inactivation of *Pseudomonas fluorescens* growth and protease production was intensively investigated with the use of Salmide®, A Sodium Chlorite-Based Oxy­halogen Disinfectant. A unique M9 media was also developed to assure sufficient protease productions with different mutants of *Pseudomonas fluorescens* as a microbioreactor. Mutations were induced by classical whole-cell mutagenesis using N-methyl-N′- nitro-N-nitrosoguanidine (NTG). A dramatic decrease occurred in protease activity when different Salmide concentrations (5, 10, and 15 ppm) were added to the growth culture followed by a complete inhibition concentration (20, 25, 50, and 100 ppm) of Salmide. However, no significant inhibition occurred once it is secreted out of cells. Some mutants were resistant and remains highly stable with high protease production under stressful conditions of Sodium Chlorite-Based Oxy­halogen. The production of the protease showed a linear correlation with the increase in incubation time using a continuous culture bioreactor system and recorded maximum protease activity after 40 h. Our findings would offer alternative antimicrobial procedures for food and industrial sectors.

## Introduction

*Pseudomonas fluorescens* is a gram-negative, rod-shaped bacterium belonging to the fluorescent pseudomonad group, a heterogeneous group of five different phenotypic characteristics (Gilligan 1995). It is an obligate aerobe that grows best at temperatures 25–30 °C (Simor et al. 1985) and is exceedingly diverse producing lipases and proteases (Montville and Winkowski 1997; Do Nascimento et al. 2022), which can lead to food spoilage.

*P. fluorescens* has been recognized as a spoilage bacteria and found to contaminate fruits, vegetables, and meat surfaces, and some food processing areas such as drains and floors (Kumar et al. 2019). Due to the creation of slime and the coagulation of proteins, these proteases cause milk to deteriorate (Law et al. 1977; Pinto 2004; Meliani and Bensoltane 2015; Morihara and Homma 2018; Zhu et al. 2019; Do Nascimento et al. 2022). Spoilage by *P. fluorescens* may attack soft rot potato and cucumber tissues (Tseng and Mount 1973; Meliani and Bensoltane 2015). *P. fluorescens* cells have been found to form biofilm on food contact surfaces, and act as reservoirs for the repeated contamination of food (Puga et al. 2016; Zhu et al. 2019), which requires several attempts to kill this spoilage bacteria using a disinfectant.

Salmide® is a series of synergistic mixtures of chlorine-containing materials that are efficient against gram-negative without using chlorine dioxide itself (Gordon 1995). It is made, in order of dominance, of water, sodium chlorite, sodium chloride, sodium chlorate, sodium borate, sodium sulfate, and hydrogen peroxide. When contacted with microorganisms, other organic matter, transition metals, or acidified, Salmide' s® oscillating reaction equilibrium is shifted to transform more of the stable chlorite and chlorate ions into the more reactive intermediate killing species (Gordon 1995). Thus, the highly reactive intermediate species created by Salmide® are more significant than the less reactive chlorite or chlorate ions or chlorine dioxide in terms of biocidal action (Gordon 1995; Lim 2003), which makes it an important antimicrobial agent that kills several foods borne pathogens (Mullerat et al. 1995; Lim 2003; Davis et al. 2010), and a promising approach for further investigation of Salmide against *P. fluorescens.*

Extracellular protease production is a well-known trait among members of *P. fluorescens* (Stanier et al. 1966; Morihara and Homma 2018). Some strains of *P. fluorescens* are considered to be a high-yield protein factories, producing medicinal, food, and industrial proteins (Landry et al. 2003; Kumar and Bhalla 2005; Sadeesh Kumar et al. 2015; Morihara and Homma 2018; Wang et al. 2022). *P. fluorescens* can be grown in bioreactors at high densities and tolerates various fermentation conditions (Schneider et al. 2005; Hu et al. 2022). The strong growth, heat stability, and high-level production of recombinant proteins make *P. fluorescens* an ideal candidate for industrial applications and cost-effective in protein recovery due to their extracellular secretions in culture medium (Landry et al. 2003; Chew et al. 2005; Fairbairn and Law 1986; Hu et al. 2022). Another advantage is that *P. fluorescens* is a possible expression host for a diverse range of recombinant proteins (Chew et al. 2005; Zhang et al. 2018; Sindhu and Manonmani 2018; Park et al. 2020; Fabia et al. 2021). So, studying *P. fluorescens* as an important tool of biotechnology is vital, especially when it extends to its mutant. Most mutations are likely to be deleterious, however, well-adapted populations form that can produce improved features and can adapt to novel environments faster than the wild types (Ilmjärv et al. 2017).

This study was undertaken to determine the biocidal activity of Salmide® against *P. fluorescens* growth and proteases. We also adopted another approach to modify M9 media to assure sufficient protease production for biotechnological applications.

## Materials and methods

### Routine cultivation and culture media

*P. fluorescens* ATCC 13525 was used as a reference in this study. The strain was grown overnight in (100 mL) of tryptic soy broth in (250 mL) Elmer flasks (TSB; Difco, Sparks, MD) at 20 °C using an orbital shaker at 200 rpm. A fresh culture media was prepared by transferring 0.1 mL culture aliquot to10 mL TSB in a test tube and incubated for 4 h at 20 °C to reach the log phase. Cell densities were measured at 600 nm using a DU730 spectrophotometer (Beckman Coulter, Pasadena, CA) along with CFU using a plate count of serial dilutions. A unique M9 media was developed in this study (1.0% glycerol, 2 ml Mole of MgSO_4_, and 1.5 ml Mole of CaC12) plus 0.01 A600 antifoams.

### Chemical agents

A stock solution of Salmide® was prepared according to the method of Gorden, G. November 1989. US patent 4,880,638. The prepared Stock was left for one month to reach the equilibrium and tested for any microbial growth. The Prepared Salmide (10 ppm) reported no growth when tested in TSB + media (Tryptic Soy Broth plus 0.5% glucose) and kept at 20 °C for one week. Each Sodium Chlorate and Sodium Chlorite was prepared alone at the same power present in the stock. Each Sodium Chlorate and Sodium Chlorite was then subjected to a 0.2 µ sterile filter membrane. All the above solutions are kept at room temperature.

### Effect of different carbon sources on *P. fluorescens* proteases and growth

A modified media was developed to achieve the maximum yield of *P. fluorescens* protease*s*. The first set of experiments was conducted to select the potential best candidates to be added to M9 media. Each Glycerol, Glucose, Sodium acetate, Sodium citrate, Alanine, Glutamine, Sucrose, and Glutamic was added to a duplicate of 50 mL M9 media's inorganic solution in 250 mL Elmer flasks at a concentration of 1% carbon sources. Inoculums were obtained from an overnight fresh TSB + culture media by harvesting cells and washing them three times using M9 buffer. Cell densities and CFU were measured as mentioned above in the cell routine section. Cells were then suspended in 10 mL of M9 media buffer. Flasks were inoculated to reach a cell density of 0.055 at 600 nm and then incubated at 20 °C for two days. The culture media was centrifuged at 15,000 rpm for 20 min at 15 °C and the supernatant was carefully discarded and tested for protease activity.

The second set of experiments was carried out by varying the concentration of selected carbon sources. The growth curve was performed in duplicate by inoculating two flasks of 100 mL of modified M9 media inoculated with a cell density of 0.055 at 600 nm. Flasks were then incubated at 20 °C for two days and samples were taken every three hours unless otherwise stated. Samples were checked for cell density and protease assay as described above.

### Hide powder azure assay

The hide powder azure assay was used for the detection of the protease quantity from *P. fluorescens* (Himelbloom, and Hassan 1985). Hide powder azure was obtained from Sigma and the procedure was modified for protease assay. The enzyme assay procedure was optimized by varying the amount of Hide powder (0.01, 0.02, and 0.03 g), rotation (static, 2 rpm, and 10 rpm), filtration (no filtration, and with 0.47µ), and pH values (4.5, 5.5, 6.5, 7.0, 7.5, 8.5, 1nd 9.0). The enzymatic reaction was carried out in duplicate by adding 3 mg of hide powder into 4.0 mL of the supernatant at pH 7.1 into a 5 mL vial. The vial was agitated and then incubated for 1.5 h at 37 °C in a water bath. Each vial was centrifuged at 10,000 rpm for 10 min and the supernatant was removed carefully and then passed through a Millipore filter pump. Absorbance at 600 nm of the filtrate was determined against the control. Controls contained everything except the supernatant. Protease activities unite was defined as absorbance at 600 per CFU/mL and measured according to our following designed equation:

All protease activity values are multiplied by a value of (10-8).

$${\text{Protease}}\,{\text{activity}} = {\text{A}}_{600} \,{\text{of}}\,{\text{enzyme}}\,{\text{assay}}\,{\text{filtrate/CFU/mL}}\,{\text{culture}}\,{\text{media}}$$

### Inhibition activity assay

Each of the following reagents, Salmide, Sodium Chlorite, and Sodium Chlorate was added to 50 mL of modified media at different concentrations as follows: 300, 200, 100, 50, 25, 15, and 0.0 ppm. Flasks were prepared in duplicates and inoculated with the suspended cells as described above and then incubated for 2 days at 20 °C. Cell densities and protease activities were measured as described above.

### Evaluation of proteases stability of *P. fluorescens* mutants

Mutations are induced by classical whole-cell mutagenesis using N-methyl-N′- nitro-N-nitrosoguanidine (NTG), which is a very effective mutagen for bacteria and yeast (Moore 1968). *P. fluorescens* were suspended in phosphate buffer (pH 6.8) with a concentration of 15 µg NTG/mL for 70 min. Mutants were used to evaluate the efficacy of antimicrobial agents on the stability of the production of proteases. Experiments were carried out as mentioned above. Cell densities and protease activities were used for evaluation.

### Inactivation mechanism of protease by Salmide

Salmide was added to 50 mL of the modified media at the following concentrations: (50, 25, 15, 10, 5, and 0 ppm). Flasks were inoculated by *P. fluorescens* (Wild type), and incubated as shown above followed by harvesting of the cell pellets and supernatants (sup 1). Both pellets and supernatants were stored in the refrigerator for the time to be used. The absorbance and protease assay were measured as described above. Cell pellets were washed three times with M9 buffer by centrifugation at 10.000 rpm for 10 min. Pellets were then suspended into 6 mL M 9 buffer in large vials and sonicated at 70 pulse*/*second until destruction was achieved after 5 min. Vials were centrifuged at 10,000 rpm for 10 min and the supernatants were removed and then assayed for protease. The supernatant obtained from the Salmide negative culture media (sup 2) was collected Salmide was added to get a series of final concentrations (50, 25, 15, 10, 5, and 0 ppm), incubated for 4 h at 20 °C, and then assayed for proteases in duplicates.

### Protease production using bioreactor

A volume of 1L M9 media was inoculated with 50 mL of one-day culture media (0.55 A_600_). Samples were taken every 3 h for growth curve study and protease activities as described above.

### Statistical analysis

Data were statistically analyzed by using the two-way variance of analysis (ANOVA) with less significant difference (L.S.D.) at (P < 0.05). Data are shown as a mean of two independent experiments with standard error (P < 0.05). Growth curve results were recorded in duplicates, and standard error was calculated for each treatment using Microsoft Excel. Data are drawn in Origin software 2017 (9.4) for data Analysis and graphing (https://www.originlab.com/Origin).

## Results

### Optimization of Hide azure enzyme assay

**Fig. 1:**
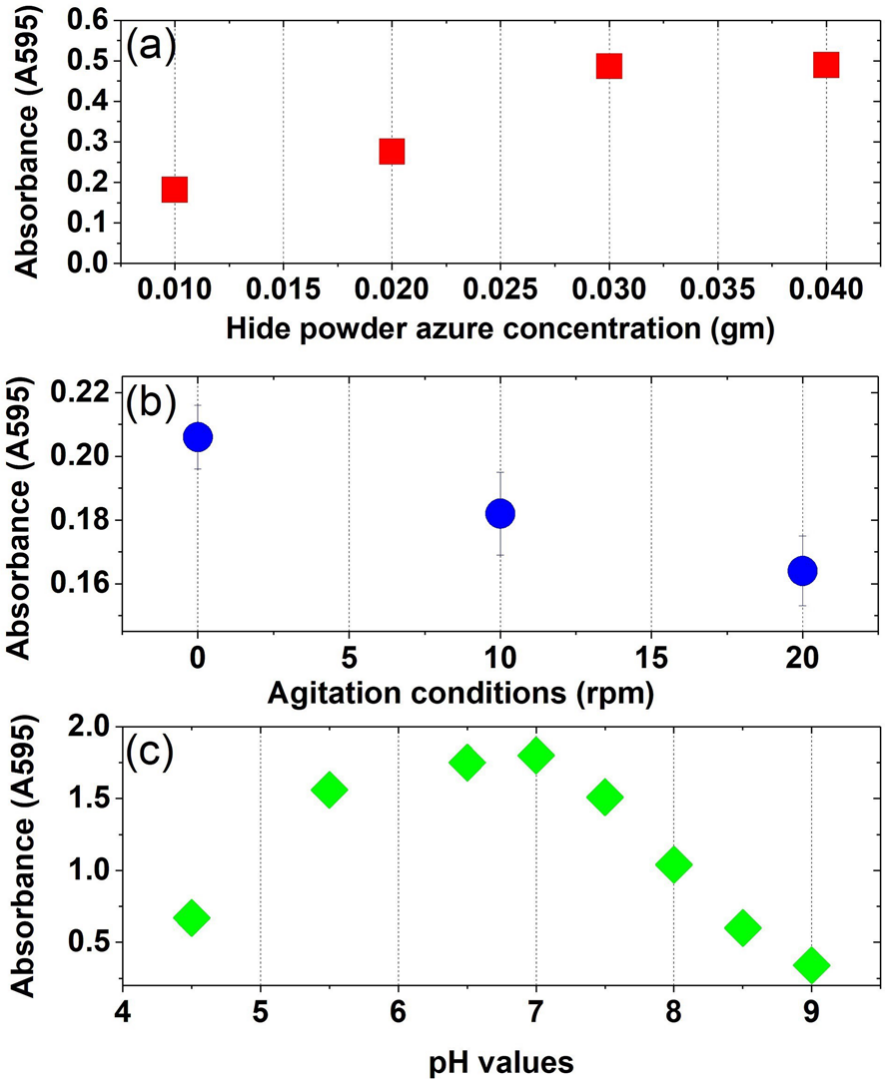
Optimization of protease enzyme activity using Hide azure enzyme assay **a** Hide Azure concentration, **b** Agitation, **c** pH values. Duplicate tubes were incubated at 37 °C for 24 h. Cell densities were measured at 600 nm using a DU730 spectrophotometer (Beckman Coulter, Pasadena, CA). Data are shown as a mean of two independent experiments with standard error (P < 0.05). Growth curve results were recorded in duplicates, and standard error was calculated for each treatment using Microsoft Excel. Data are drawn in Origin software 2017 (9.4) for data Analysis and graphing (https://www.originlab.com/Origin)

Optimization procedures of concentration, agitation, and pH values in Hide azure enzyme assay showed different protease activity patterns. The maximum concentration of the protease activity (0.04) gm of Hide powder azure was recorded with A_600_ of (0.49 O.D), while lower concentrations reported lower enzyme absorbance ranging from (0.1–0.4 O.D) (Fig. 1a). Static conditions increased the protease activity (0.206 O.D), while agitation at (10–20) rpm showed lower protease activity (Fig. 1b). Optimization of pH values showed that pH 4.5 recorded the highest protease activity (0.67 O.D). Higher pH values showed a gradual decrease in the protease activity (Fig. 1c).

### Effect of different carbon sources on *P. fluorescens* proteases and growth

**Fig. 2:**
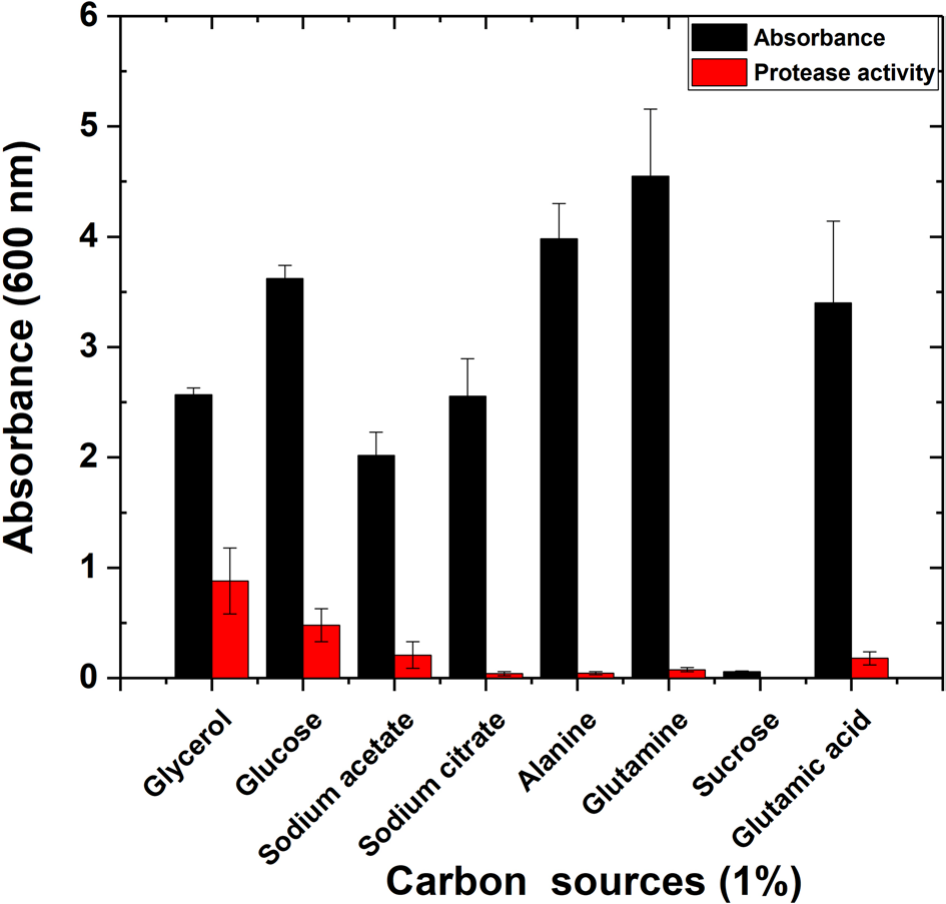
Optimization of the growth and protease production by *P. fluorescens* strain. Several carbon sources were used at a concentration of (1%) in M9 media including glycerol, glucose, sodium acetate, sodium citrate, alanine, glutamine, sucrose, and Glutamic acid substrates. Duplicate tubes were incubated at 37 °C for 24 h. Negative and positive controls were performed. Cell densities were measured at 600 nm using a DU730 spectrophotometer (Beckman Coulter, Pasadena, CA) along with CFU using plate count of serial dilutions. Data are shown as a mean of two independent experiments with standard error (P < 0.05). Growth curve results were recorded in duplicates, and standard error was calculated for each treatment using Microsoft Excel. Data are drawn in Origin software 2017 (9.4) for data Analysis and graphing (https://www.originlab.com/Origin)

Optimization of carbon sources was conducted showing the maximum protease production in the case of glycerol and glutamate substrates with (0.881 O.D) and (0.5 O.D), respectively. Higher yields of protease activity were recorded in the following order by using glucose, sodium citrate, and alanine (Fig. 2). The lowest protease activity (0.3 O.D) and (0.1 O.D) were determined with sodium acetate and glutamic acid substrates. *P. fluorescens* failed to consume sucrose; neither protease activity nor growth was noticed.

### Effect of different concentrations of glycerol, MgSO_4_, and CaCl_2_ on the yield of *P. fluorescens* proteases and growth

**Fig. 3:**
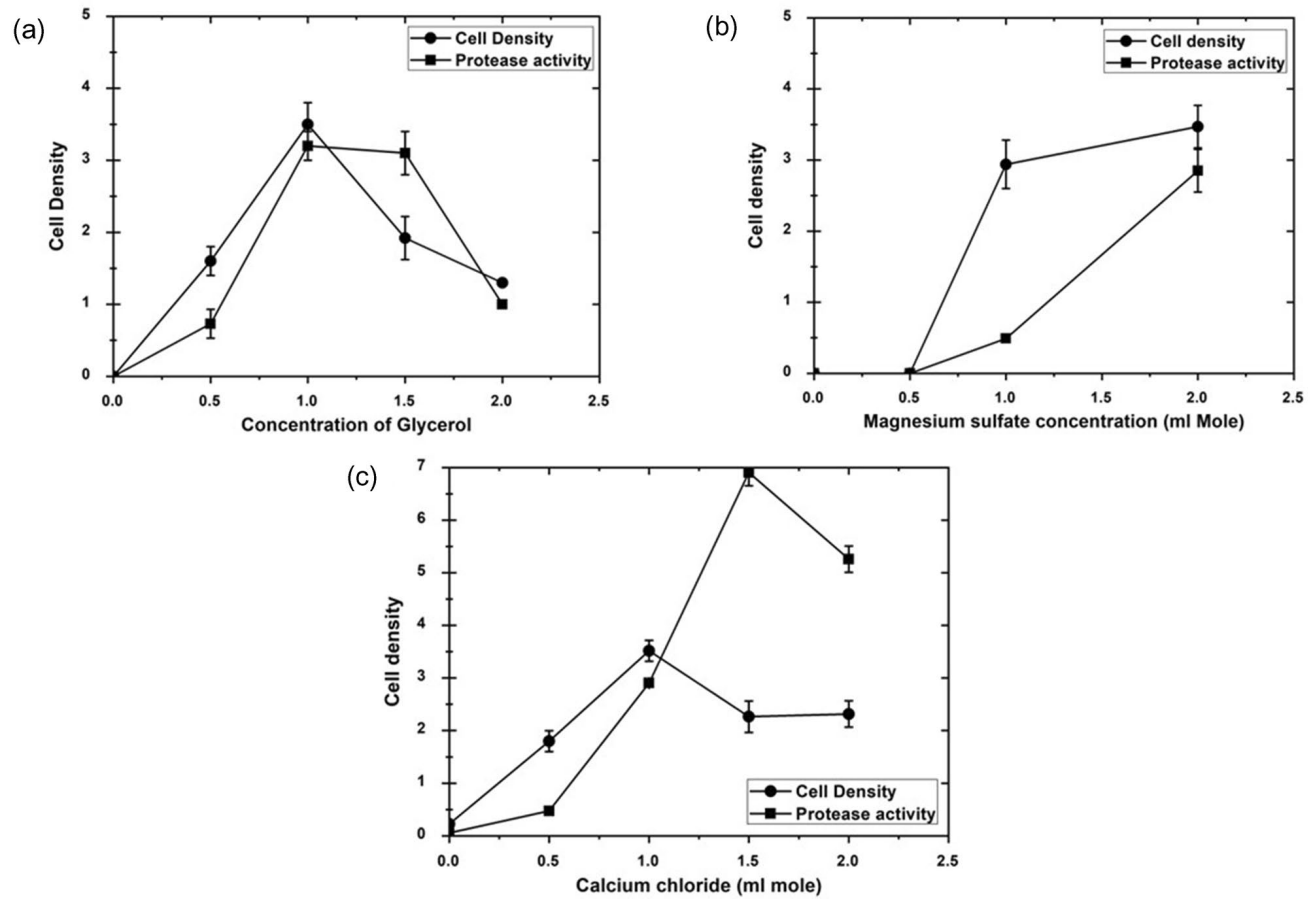
Growth and protease activity by *Pseudomonas fluorescence* in M9 media. Concentrations of M9 media components were added as follows: **a** Glycerol (0, 0.5, 1.0, 1.5 and 2.0 mL mole), **b** Magnesium sulfate concentrations (0, 0.5, 1.0, 1.5 and 2.0 mL mole), **c** Calcium chloride (0,0.5, 1.0, 1.5 and 2.0 mL mole). Duplicate tubes were incubated at 37 °C for 24 h. Negative and positive controls were performed, and cell densities were measured at 600 nm using a Du730 spectrophotometer (Beckman Coulter, Pasadena, CA) along with CFU using plate count of serial dilutions. Data are shown as a mean of two independent experiments with standard error (P < 0.05). Growth curve results were recorded in duplicates, and standard error was calculated for each treatment using Microsoft Excel. Data are drawn in Origin software 2017 (9.4) for data Analysis and graphing (https://www.originlab.com/Origin)

Glycerol carbon source showed the highest yield of protease as well as a significant yield of cell density. Optimization of glycerol, MgSO_4_, and CaCl_2_ concentrations indicated that protease production was maximum at (1.0) mL mole of glycerol, stationary at a concentration of (1.5) mL mole, and the least production was recorded at (2.0) mL mole (Fig. 3a). As for magnesium sulfate, it increased the cell density by increasing the concentration till it reaches a maximum value of cell density and protease production at (2) mL mole (Fig. 3b), and in the case of calcium chloride (Fig. 3c) low activity of protease production was recorded at (0.5) mL mole, followed by an increase at (1.0) mL mole and it reaches maximum protease activity concentration at 1.5 mmol and then static activity occurs at 2.0 mL mole concentration. The maximum protease production was obtained at 1% glycerol, 2 mL Mole of MgSO_4_, and 1.5 mL Mole of CaCl_2_ in M9 media buffer (Fig. 3a–c).

### Biocidal activities and inactivation of proteases in the presence of different concentrations of antimicrobial agents

**Fig. 4:**
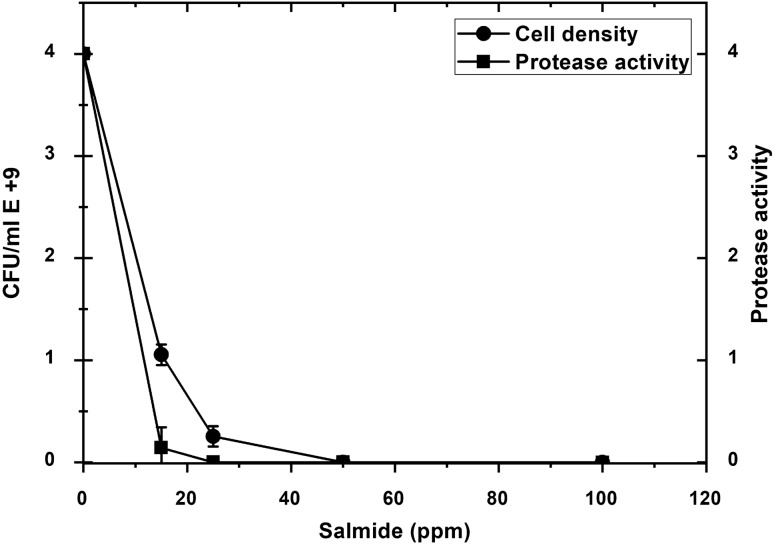
Effect of Salmide on the growth and protease activity of *P. fluorescens*. Several concentrations of Salmide (0, 15, 25, 50, 100, 200 and 300 ppm) were added to 50 mL of modified media, Flasks were prepared in duplicates and inoculated with suspended cells, and then incubated for 2 days at 20 °C. Cell densities and protease activities were measured at 600 nm using a Du730 spectrophotometer (Beckman Coulter, Pasadena, CA) along with CFU using plate count of serial dilutions. Data are shown as a mean of two independent experiments with standard error (P < 0.05). Growth curve results were recorded in duplicates, and standard error was calculated for each treatment using Microsoft Excel. Data are drawn in Origin software 2017 (9.4) for data Analysis and graphing (https://www.originlab.com/Origin)

**Fig. 5:**
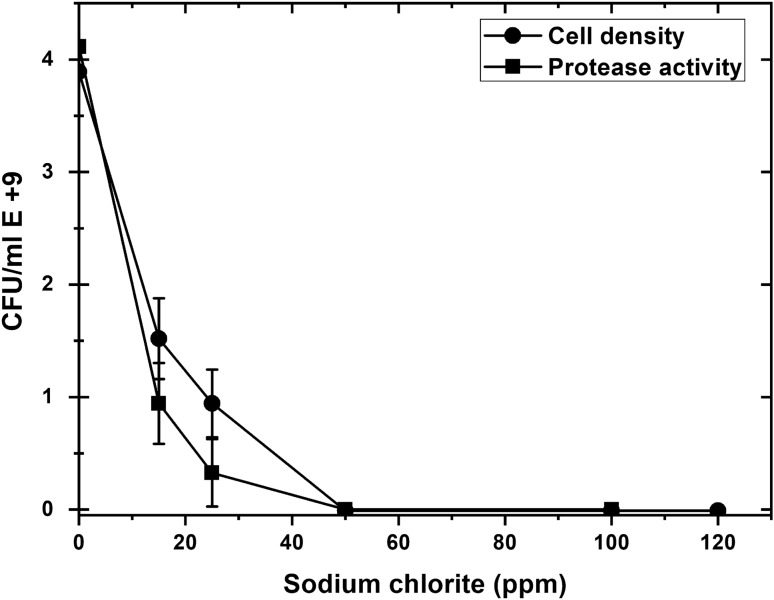
Effect of Sodium Chlorite on the growth and protease activity by *P. fluorescens.* Several concentrations of Sodium Chlorite (0, 15, 25, 50, 100, 200 and 300 ppm) were added into 50 mL of modified media. Flasks were prepared in duplicates and inoculated with the suspended cells and then incubated for 2 days at 20 °C. Cell densities and protease activities were measured at 600 nm using a Du730 spectrophotometer (Beckman Coulter, Pasadena, CA) along with CFU using plate count of serial dilutions. Data are shown as a mean of two independent experiments with standard error (P < 0.05). Growth curve results were recorded in duplicates, and standard error was calculated for each treatment using Microsoft Excel. Data are drawn in Origin software 2017 (9.4) for data Analysis and graphing (https://www.originlab.com/Origin)

**Fig. 6:**
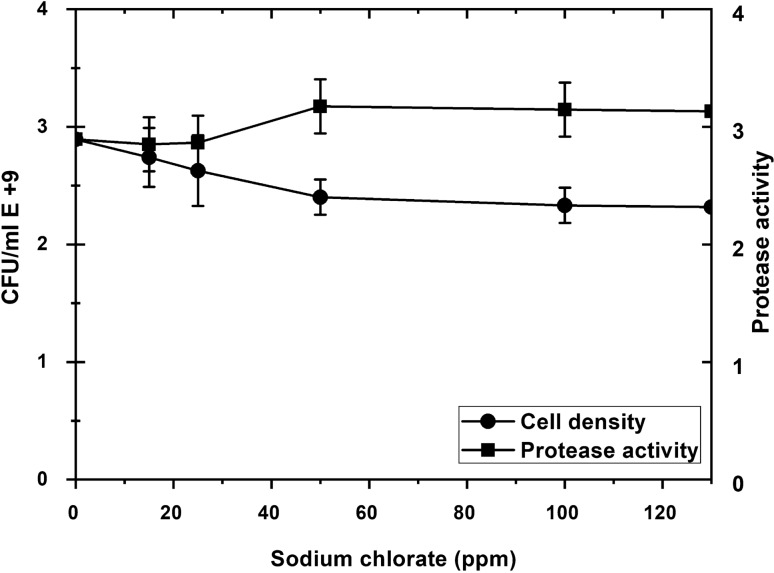
Effect of Sodium Chlorate on the growth and protease activity of *P. fluorescens.* Several concentrations of sodium chlorate (0, 15, 25, 50, 100, 200 and 300 ppm) were added to 50 mL of modified media, Flasks were prepared in duplicates and inoculated with the suspended cells, and then incubated for 2 days at 20 °C. Cell densities and protease activities were measured at 600 nm using a Du730 spectrophotometer (Beckman Coulter, Pasadena, CA) along with CFU using plate count of serial dilutions. Data are shown as a mean of two independent experiments with standard error (P < 0.05). Growth curve results were recorded in duplicates, and standard error was calculated for each treatment using Microsoft Excel. Data are drawn in Origin software 2017 (9.4) for data Analysis and graphing (https://www.originlab.com/Origin)

The biocidal and inhibition activity of different concentrations of Salmide, Sodium Chlorite, and Sodium Chlorate was tested against *P. fluorescens* growth and protease activity. A sharp drop in cell density, as well as protease activity, occurred with the increase in Salmide concentrations. At a concentration of 50 ppm, neither protease activity nor growth was observed reporting complete destruction of the cells (Fig. 4). Similar results were obtained when cells were grown in the presence of Sodium Chlorite (Fig. 5). A slight decline in cell density occurred when cells were grown at (15, 25, and 50 ppm) of Sodium Chlorate resulting in a decrease in protease activity, followed by a steady growth of cells. Protease activity was observed at a concentration between 100 and 300 ppm (Fig. 6).

### Inactivation mechanism of protease by Salmide

**Fig. 7:**
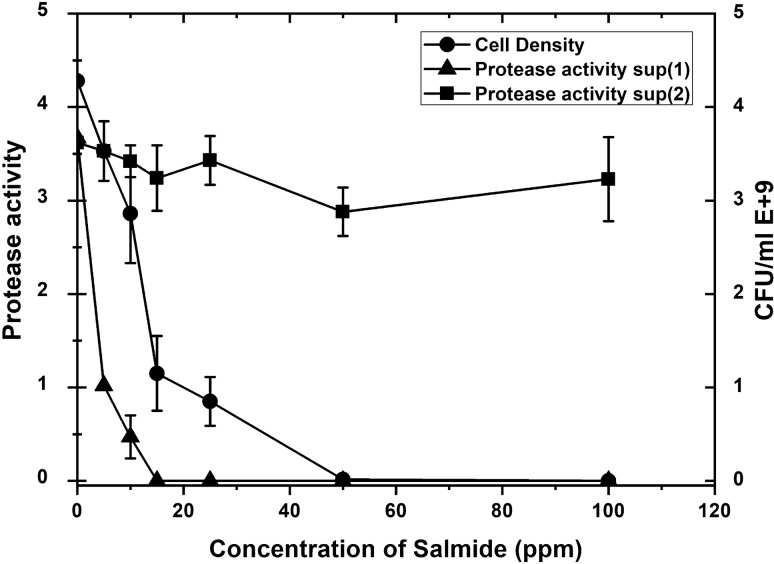
Effect of Salmide on the growth and protease activity of *P. fluorescens* supernatants (1 and 2). Several concentrations of Salmide (0, 5, 10, 15, 20, 25, 50, and100 ppm) were added to 50 mL of modified media, Flasks were prepared in duplicates and inoculated with the suspended cells of by *P. fluorescens* (Wild type)and then incubated for 2 days at 20 °C followed by harvesting of the cell pellets and supernatants (sup 1). Both pellets and supernatants were stored in the refrigerator for the time to be used. The absorbance and protease assay were measured as described above. Cell pellets were washed three times with M9 buffer by centrifugation at 10.000 rpm for 10 min. Pellets were then suspended into 6 mL M 9 buffer in large vials and sonicated at 70 pulse*/*second until destruction was achieved after 5 min. Vials were centrifuged at 10,000 rpm for 10 min and the supernatants were removed and then assayed for proteases. The supernatant obtained from the Salmide negative culture media (sup 2) was collected Salmide was added to get a series of final concentrations (50, 25, 15, 10, 5, and 0 ppm), incubated for 4 h at 20 °C, and then assayed for proteases in duplicates. Cell densities and protease activities were measured at 600 nm using a Du730 spectrophotometer (Beckman Coulter, Pasadena, CA) along with CFU using plate count of serial dilutions. Data are shown as a mean of two independent experiments with standard error (P < 0.05). Growth curve results were recorded in duplicates, and standard error was calculated for each treatment using Microsoft Excel. Data are drawn in Origin software 2017 (9.4) for data Analysis and graphing (https://www.originlab.com/Origin)

The supernatant (sup 1), which was obtained from cell culture growth with different concentrations of Salmide, showed a dramatic decrease in protease activity with different Salmide concentrations (5, 10, and 15 ppm) added to the growth culture followed by a complete inhibition concentration (20, 25, 50, and 100 ppm) of Salmide (Fig. 7). When salmide was added to salmide negative supernatant (sup 2), a nonsignificant inhibition of protease activity was observed at different concentrations (5, 10, 15, 25, 50, and 100) of Salmide. This observation indicated that Salmide inhibits the production of proteases (sup 1) in culture media, however, no significant inhibition occurred once it is secreted out of cells (sup 2). Cells density showed a sharp inhibition at (15 ppm) of Salmide then a gradual decrease of cell density was recorded from (20 ppm) to (50 ppm). Complete inhibition of cell density was recorded at (50 ppm) Salmide and followed by null steady values.

### Evaluation of proteases stability of *P. fluorescens* mutants

Random mutations were generated by N-methyl-N′- nitro-N-nitrosoguanidine (NTG), among the mutants. F4-b, Fm1-b, Fm7-b mutants showed higher activities of growth and protease activity than the other mutants shown in (Table 1). Mutant F4-b showed the highest protease activity with a 1.071 CFU/protease activity ratio. Fm1-b showed the highest protease activity values, in contrast, mutants Fm1-c and Fm1-d showed no protease activity. As for Fm2-b, Fm3-b, Fm4-b, Fm5-b, Fm6-b, and Fm7-b mutants, they showed the highest protease activity ratio while Fm2-d, Fm3-d, Fm4-d, Fm5-d, Fm6-d, and Fm7-d mutants showed no protease activity. Mutant Fm8-d showed the highest protease activity, while Fm8-b presented the lowest activity in Fm8 mutant group (Table 1).

### Production of protease using a bioreactor via continuous culture system

**Fig. 8:**
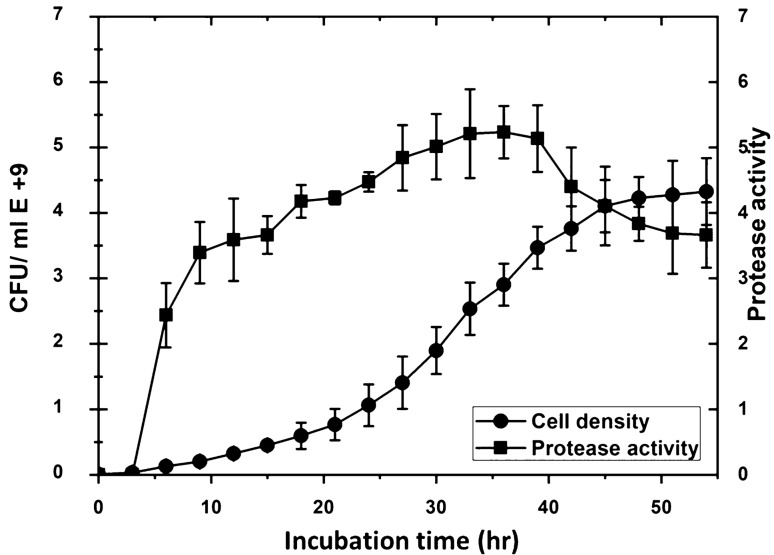
The effect of incubation time on the growth and protease production by *P. fluorescens.* A volume of 1L M9 media was inoculated with 50 mL of one-day culture media (cell density 0.55 A_600_). Samples were taken every 3 h for growth curve study and protease activity. Cell densities and protease activities were measured at 600 nm using a Du730 spectrophotometer (Beckman Coulter, Pasadena, CA) along with CFU using plate count of serial dilutions. Data are shown as a mean of two independent experiments with standard error (P < 0.05). Growth curve results were recorded in duplicates, and standard error was calculated for each treatment using Microsoft Excel. Data are drawn in Origin software 2017 (9.4) for data Analysis and graphing (https://www.originlab.com/Origin)

**Fig. 9:**
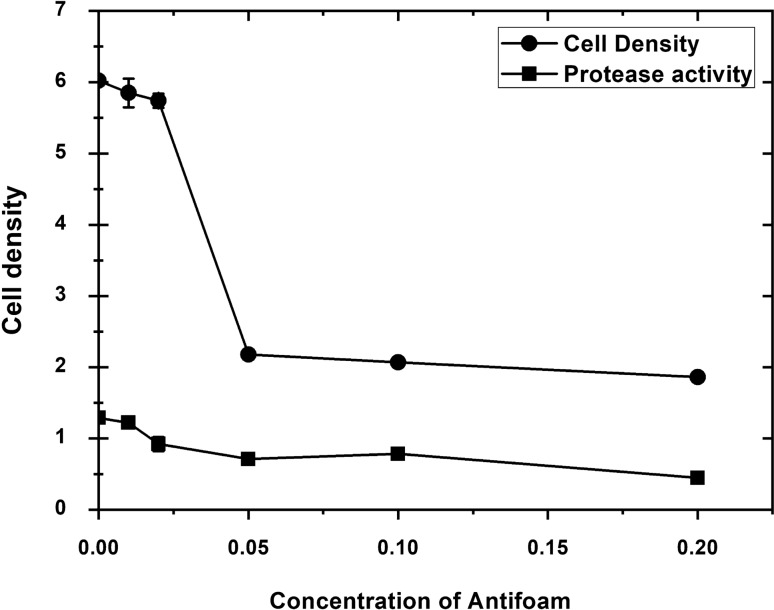
Effect of Antifoam concentration on protease production by *P. fluorescens.* A volume of 1L M9 media was inoculated with 50 mL of one-day culture media (cell density 0.55 A). Several concentration of antifoam were used at a concentration of (0.00, 0.01, 0.02, 0.05, 0.10, 0.20 gm). Cell densities and protease activities were measured at 600 nm using a Du730 spectrophotometer (Beckman Coulter, Pasadena, CA) along with CFU using plate count of serial dilutions. Data are shown as a mean of two independent experiments with standard error (P < 0.05). Growth curve results were recorded in duplicates, and standard error was calculated for each treatment using Microsoft Excel. Data are drawn in Origin software 2017 (9.4) for data Analysis and graphing (https://www.originlab.com/Origin)

The bioreactor was optimized to reach the highest threshold of cell density and the protease produced by *P. fluorescens* strain (Wild type). The production of the protease showed a linear correlation with the increase in incubation time. The maximum protease activity was recorded after (40 h) of incubation (Fig. 8). The protease production increases as the cell density increases during the log phase and the stationary phase when the cell grows with shaking incubation. The maximum protease was obtained at the end of the stationary phase and then become steady during the lag phase. This indicates that protease activity could be related to cell density and incubation time-dependent on the growth media. The continuous culture system required adding antifoam agents. The addition of different densities of antifoams to both shake flask and bioreactor cultures of *P. fluorescens* altered the total yield of cell densities and protease production. It looks like antifoams impact the net cell densities of the culture media, however, keeping cells young and secreting constant significant amounts of proteases. Antifoaming agents may therefore have specific effects on the growth metabolism and yield characteristics of the cultures. Results showed that cell densities decreased as antifoam density increased to A_600_ of 0.05 and then become steady at A_600_ of 0.10 to 0.20 as shown in (Fig. 9).

## Discussion

In this manuscript, we’ve extensively investigated the influences of Salmide as a unique antimicrobial agent against *P. fluorescens* growth and protease production. Several growth factors were optimized for maximal protease production and biotechnological application purposes. These operation conditions such as aerobic fermentation, media type, oxygen availability, and pH values were found to dramatically influence metabolic pathways and change metabolic fluxes to increase the protease yield (Çalık et al. 2002). Indeed, the media components are important tools for bioprocess medium design and biotechnological applications (Çalık et al. 2001). In this study, the highest protease production was obtained via optimization of the microbioreactor conditions (agitation, pH, and nutrient concentration).

Agitation and aeration are effective parameters for growth and protease production by psychrotrophs. We’ve noticed that static conditions increased the protease activity produced by *P. fluorescens,* while agitation values at (10–20) rpm showed lower proteases activities. Similarly, it was reported that agitation speed alters protease yield by *Pseudomonas putida*, and several protease yield values (97, 283, 431, and 49 U mL^−1^) were achieved at (50, 100, 150, and 250 RPM), respectively (Singh et al. 2011). Additionally, it was confirmed that speeds above 200 rpm led to a decrease in enzyme production due to the mechanical damage of bacterial cells and enzymes (Potumarthi et al. 2007).

The pH value is a key factor affecting bacterial growth and protease production (Ahangari et al. 2021). Our results showed that pH 7 recorded the highest protease activity and then gradually decrease with higher pH values. In contrast, another study reported that maximum protease production at pH 9.0 by *P. fluorescens* then declined with higher alkaline values above pH 9 (Kalaiarasi and Sunitha 2009). The obtained results coincide with Kumar et al. (2002) who reported that protease production via *Bacillus *sp. strain S4 was maximum at pH 7 and the yield of protease was increased at pH 7 (Gu et al. 2019). Comparable relevant studies indicated that pH stresses can impact the structures of microbial proteases as it affects many enzymatic processes and the transport of compounds across the cell membrane (Awad et al. 2020; Buchholz et al. 2020). Thus, hydrogen bonds and hydrophobic interactions are the major forces that promote the binding of the protease and myosin light chain and can also accelerate the catalytic hydrolysis of the substrate by the protease (Cao et al. 2020).

Nutritional requirements for optimized protease production by *P. fluorescens* were also investigated. Optimization of carbon sources was conducted showing maximum protease production in the case of glycerol and glutamate substrates, while the lowest protease activity was determined in the case of sodium acetate and glutamic acid substrates. Alternatively, it was reported that maximum protease activity was achieved with sorbitol as the sole carbon source, followed by starch and lactose at pH 7.0 and 37 °C (Abd Rahman et al. 2005). Otherwise, it was reported that maltose was found to support protease production (Sudharshan et al. 2007). Our study agrees with (Abd Rahman et al. 2005), showing that the sucrose carbon source was completely unsuitable for enzyme production by *Pseudomonas* species.

A metal ion in media is an important factor that affects enzyme production. The maximum protease production was obtained at 2 mL Mole of MgSO_4_, and 1.5 mL Mole of CaCl_2_ in M9 media buffer. On the other hand, Abd Rahman et al. (2005) reported that CaCl_2_ at a concentration of 0.2% inhibits protease production followed by CuSO_4_ and BaCl_2_. Correspondingly, it was reported that *Pseudomonas* proteases usually require Ca^2+^ or Zn^2+^ as cofactors (Sørhaug and Stepaniak 1997; Liao and McCallus 1998; Jankiewicz et al. 2010). Calcium is probably a cofactor for this enzyme, since it has a calcium-binding domain in its structure, like other proteases from psychrotrophic bacteria, suggesting that this enzyme is a metalloprotease that requires metallic ions in the active site to maintain its structure and stability (Fox and McSweeney 1998).

Microorganisms and proteases play an important role in the formation of tenderness, changing the flavor, and quality of fermented meat products, milk and dairy products (Sorapukdee et al. 2020; Fadda et al. 2010; Zhou et al. 2013). In this study, we investigated the inhibition of *P. fluorescence* growth and protease production by Salmide, A Sodium Chlorite-Based Oxy­halogen disinfectant. We synthesized Salmide resulting in a stabilized redox-buffered equilibrium of specific biocidal intermediates (Gordon 1989a). The chemical formulation of Salmide results in a pseudo-oscillating chemical reaction in which chlorite, chloride, and chlorate ions are predominant species, while superoxide and hypochlorite ions are formed in smaller amounts along with reactive intermediates (Cl_2_O_2_, HCl_2_O_2_, and Cl_2_O_4_) and minimal amounts of chlorine dioxide (Gordon 1989b). The redox-buffering effect at alkaline pH is responsible for its stability in the absence of organic matter. When it contacts micro-organisms and other organic material, Salmide’s pseudo-oscillating reaction equilibrium is shifted to convert more of the stable chlorite and chlorate ions into the more reactive bactericidal intermediates (Gordon 1989a; b). We tested the biocidal and inhibition activity of different concentrations of Salmide, Sodium Chlorite, and Sodium Chlorate against *P. fluorescens* growth and protease activity. We observed a sharp drop in cell density, as well as protease activity, which occurred with the increase of Salmide concentrations indicating the destruction of the cells. Our results indicated that Sodium Chlorite inhibits the *Pseudomonas fluorescence* growth with a decrease in protease activity followed by a steady state. Similarly, it was known that Sodium Chlorite (SC) is a chemical approved by the Food and Drug Administration (FDA) for the decontamination of poultry and red meat carcasses (CFR 1998; Hajmeer et al. 2004; Kemp 2000). Sodium Chlorite (SC) is a well-known antimicrobial agent, due to its strength as an oxidizing agent. Under acidic conditions, it can generate chlorine dioxide gas, which is also a powerful oxidizing agent and has been used to sterilize equipment and food preparation surfaces, prevent, and remove biofilms, bleach paper, textiles, flour, and disinfect water (Lu et al. 2006; Luo et al. 2011). Moreover, recent studies have shown that SC also effectively inhibits the enzymatic browning of fresh-cut apples (Lu et al. 2007). Therefore, SC has a dual effect on browning inhibition and pathogen inactivation (He et al. 2008).

Additionally, Sodium Chlorite is therefore one of several antimicrobials effective in post-chill interventions for reducing pathogens in poultry (Oyarzabal 2005). It was hypothesized that the mode of action of ASC derives from the uncharged chlorous acid, which is formed by the acidification of chlorite. Chlorous acid gradually decomposes to form chlorate ions, chlorine dioxide, and chloride ions. These reactive intermediates are highly oxidative with broad-spectrum germicidal activity (FDA 2007; Warf 2001); ; . Chlorous acid is also able to penetrate bacterial cell walls. This ability is thought to facilitate proton leakage into cells, which increases energy use by the cells to maintain homeostasis (Warf 2001).

We optimized conditions in the bioreactor to reach the highest threshold of cell density and the protease produced by *P. fluorescens* strain. We noticed that the protease production increases as the cell density increases during the log phase and reaches its maximum production at the end of the stationary phase followed by a steady status during the lag phase. This indicates that protease activity is related to cell density and incubation time-dependent on the growth media. We affirmed that adding antifoams to both shake flask and bioreactor cultures of *P. fluorescens* altered the total yield of protease production. A possible explanation is that the presence of the antifoam decreases the total amount of protease being produced and secreted per cell or that it decreases the density of the culture. It was reported that the production of degradative enzymes normally occurs in the late logarithmic phase of growth when the cell density is high. This production occurs through a phenomenon called quorum sensing. It normally involves the activation of specific genes at high cell densities in response to chemical signals released by *P. aeruginosa*. It was confirmed that once the cell densities and autoinducers have reached a certain threshold level, generally in the late logarithmic phase, the expression of genes encoding exoproteins and secretion systems is induced (Swift et al. 10), which can discuss the protease production results in our study. More detailed investigations will be conducted in future work to explore the mechanism of Quorum sensing and protease activity expression as it was a limitation in this study.

This work successfully monitors the *P. fluorescens* growth and protease production using Salmide® in a unique M9 media. Our results also shed the light on using an industrial microorganism such as *P. fluorescens* as a microbioreactor. We introduced different optimization methodologies using M9 media to increase the protease yield and enhance the micro bioreactor's performance. Future work would be done to pinpoint the best protocol for controlling *P. fluorescens* growth and protease production in food samples. This study will also establish full awareness and encourages the utilization of this disinfectant to avoid technical problems in the food industry and support the public health sector.

**Table 1:** Evaluation of proteases stability of *P. fluorescens* mutants

Mutants	CFU	Protease activity	CFU/Protease activity	Standard errors
F4-a	2.061	0.94	0.455	0.012
F4-b	0.876	0.938	1.071	0.023
F4-c	0.826	1.094	0.654	0.022
F4-d	4.96	1.534	0.309	0.10
Fm1-a	0.77	0.327	0.425	0.013
Fm1-b	1.116	0.682	0.611	0.014
Fm1-c	4.265	0.000	0.00	0.00
Fm1-d	1.652	0.00	0.00	0.00
Fm2-a	0.988	0.22	0.223	0.011
Fm2-b	1.568	0.444	0.283	0.022
Fm2-c	4.29	0.00	0.00	0.00
Fm2-d	1.652	0.00	0.00	0.00
Fm3-a	0.829	0.078	0.094	0.004
Fm3-b	4.855	1.362	0.281	0.007
Fm3-c	04.915	0.00	0.00	00
FM3-d	1.624	0.00	0.00	00
Fm4-a	0.978	0.281	0.287	0.005
Fm4-b	0.526	0.167	0.317	0.013
Fm4-c	4.915	0.00	0.00	0.00
Fm4-d	3.212	0.00	0.00	000
Fm5-a	0.633	0.185	0.292	0.04
Fm5-b	0.869	0.326	0.375	0.03
Fm5-c	4.385	0.00	0.00	0.00
Fm5-d	3.915	0.00	0.000	0.00
Fm6-a	0.383	0.052	0.136	0.001
Fm6-b	0.791	0.173	0.166	0.002
Fm6-c	3.365	0.00	0.00	0.00
Fm6-d	4.515	0.00	0.00	0.00
Fm7-a	0.644	0.107	0.166	0.002
Fm7-b	0.934	0.52	0.557	0.03
Fm7-c	4.63	0.00	0.00	0.0
Fm7-d	2.52	0.00	0.00	0.0
Fm8-a	0.450	0.084	0.187	0.013
Fm8-b	0.828	0.258	0.311	0.02
Fm8-c	0.362	0.062	0.171	0.012
Fm8-d	0.606	0.219	0.361	0.02

The colony forming unit (CFU) and the protease activity are the mean of two independent experiments with standard error (P < 0.05) and standard error was calculated for each treatment using Microsoft Excel

*CFU* colony-forming unit

## Data Availability

The datasets generated during the current study are available from the corresponding author on reasonable request.
